# *CgGCS*, Encoding a Glucosylceramide Synthase, Is Required for Growth, Conidiation and Pathogenicity in *Colletotrichum gloeosporioides*

**DOI:** 10.3389/fmicb.2019.01016

**Published:** 2019-05-21

**Authors:** Yimei Huang, Bin Li, Jian Yin, Qiaosong Yang, Ou Sheng, Guiming Deng, Chunyu Li, Chunhua Hu, Tao Dong, Tongxin Dou, Huijun Gao, Fangcheng Bi, Ganjun Yi

**Affiliations:** ^1^Institute of Fruit Tree Research, Guangdong Academy of Agricultural Sciences, Guangzhou, China; ^2^Key Laboratory of South Subtropical Fruit Biology and Genetic Resource Utilization, Ministry of Agriculture, Guangzhou, China; ^3^Key Laboratory of Tropical and Subtropical Fruit Tree Research, Guangdong Province, Guangzhou, China; ^4^College of Horticulture and Forestry Sciences, Huazhong Agricultural University, Wuhan, China; ^5^College of Life Sciences, South China Agricultural University, Guangzhou, China; ^6^School of Life Sciences, Sun Yat-sen University, Guangzhou, China

**Keywords:** *Colletotrichum gloeosporioides*, glucosylceramide, GCS conidiogenesis, growth rate, pathogenicity

## Abstract

Fungal glucosylceramide plays important role in cell division, hyphal formation and growth, spore germination and the modulation of virulence and has recently been considered as target for small molecule inhibitors. In this study, we characterized CgGCS, a protein encoding a glucosylceramide synthase (GCS) in *Colletotrichum gloeosporioides*. Disruption of *CgGCS* resulted in a severe reduction of mycelial growth and defects in conidiogenesis. Sphingolipid profile analysis revealed large decreases in glucosylceramide production in the mutant strains. Pathogenicity assays indicated that the ability of the Δ*CgGCS* mutants to invade both tomato and mango hosts was almost lost. In addition, the expression levels of many genes, especially those related to metabolism, were shown to be affected by the mutation of CgGCS via transcriptome analysis. Overall, our results demonstrate that *C. gloeosporioides* glucosylceramide is an important regulatory factor in fungal growth, conidiation, and pathogenesis in hosts.

## Introduction

*Colletotrichum gloeosporioides* is a pathogen that causes anthracnose in a broad range of plant hosts, including mango, avocado, yam cassava and strawberry, and is one of the most important postharvest pathogenic fungi in fruit (Perfect et al., 1999; Cannon et al., 2000; Hyde et al., 2009). Postharvest pathogens usually cause big decay losses of postharvest fruits during postharvest handling, storage, transportation and marketing (Nunes, 2012).

Glucosylceramide (GlcCer) is composed of a sphingoid backbone, a fatty acid, and a glucose moiety. GlcCer exist in membrane of plants, fungi and animals and are not present in bacteria and in some eukaryotes, such as *Saccharomyces cerevisiae* (Del Poeta et al., 2014). Except functioning as a membrane component, GlcCer is also involved in spore germination, hyphal growth, morphogenesis, virulence, and differentiation in fungi via its regulation of the physical properties of membranes in several human and plant pathogens (Levery et al., 2002; Warnecke and Heinz, 2003; da Silva et al., 2004; Rittershaus et al., 2006; Ramamoorthy et al., 2007; Rittenour et al., 2011; Zhu et al., 2014). The Glucosylceramide synthase (GCS) catalyzed the final step of GlcCer synthesis by transferring a glucosyl residue to sphingoid backbone. In *Aspergillus nidulans* and *Aspergillus fumigatus*, inhibition of GlcCer synthesis using a GCS inhibitor strongly affected spore germination and hyphal growth (Levery et al., 2002). In the human pathogen *Cryptococcus neoformans*, the deletion of GCS causes complete loss of virulence in a mouse model (Rittershaus et al., 2006). In the Citrus pathogen *Penicillium digitatum*, a GCS1 mutant exhibited an apparent decrease in virulence (Zhu et al., 2014). However, in *Fusarium graminearum*, GCS1 is not required for pathogenicity, but it does affect conidial morphology and hyphae growth (Ramamoorthy et al., 2007), suggesting that it plays different roles in different fungi.

There is much evidence to support the critical role of GlcCer in the pathogenicity of many different fungi (Del Poeta et al., 2014). Moreover, fungal GlcCer is structurally different from the mammalian homolog (Del Poeta et al., 2014). Thus, monoclonal antibodies to fungal GlcCer have been used to inhibit hyphae growth (Rodrigues et al., 2000; Nimrichter et al., 2004) and prevent *Cryptococcal* infection in mice (Rodrigues et al., 2000). In addition, defensins, well-known potent antimicrobial peptides that have been isolated from plants or insects, can interact with fungal GlcCer and cause growth arrest *in vitro* or *in vivo* (Thevissen et al., 2004, 2007, 2012; Aerts et al., 2008; Tavares et al., 2008). Overall, fungal GlcCer has served as an ideal target for new antimicrobials (Nimrichter and Rodrigues, 2011).

Though previous research reports have indicated that GlcCer antibodies can block the differentiation of conidia into mycelia in *C. gloeosporioides* (da Silva et al., 2004), the true role played by GlcCer in this important postharvest pathogen is still not clear. In the present work, we identified the main role of GCS, the gene that encodes GlcCer synthase in *C. gloeosporioides*, which is an important agent that causes anthracnose in postharvest fruits. The results suggest that GCS is key regulator of growth, sporulation and virulence in *C. gloeosporioides.*

## Materials and Methods

### Fungal Strains, Media and Growth Conditions

The WT strain Cg-14 isolate was obtained from a decaying avocado fruit (*Persea americana* CV. Fuerte) in Israel (Yakoby et al., 2001) and has been routinely cultured on M_3_S media (Tu, 1985). The M_3_S medium contains (per L) 2.5 g MgSO_4_.7H_2_O, 2.7 g KH_2_PO_4_, 1 g peptone, 1 g yeast extract, 10 g sucrose, 250 mg chloramphenicol, and 2% (w/v) agar. The cultures were incubated in M_3_S medium (without agar) at 150 rpm for 3 days at room temperature and were collected by vacuum filtration through a sterile funnel setted with filter paper. The hyphal mat was washed twice with 40 ml of sterile distilled water. The washed mycelia were resuspended in 50 ml of a fresh second medium.

### Vector Construction and Transformation

To prepare the GCS-knockout construct, 509 bp of the 5′ flanking fragment and 568 bp of the 3′ flanking fragment of the coding region were amplified from the full GCS gene from *C. gloeosporioides* (TCONS_00007676, contig01842). The 5′ and 3′ fragments of GCS gene were amplified with the primer sets attBGCS_5′F + attBGCS_5′R and attBGCS_3′F + attBGCS_3′R (Supplementary Table S1), respectively. The Gateway system was used to build the construct as previously described (Shafran et al., 2008). The *Not* I-digested plasmid fragment was used to generate the GCS knockout mutant. For the complementation construct, a 3431 bp fragment of genomic DNA from the GCS gene that included the native promoter (1263 bp) and terminator (540 bp) sequences was cloned into the pDEST phleo vector using the primer set attB_GCS_compF and attB_GCS_compR (Supplementary Table S1).

Fungal transformation was performed using PEG-mediated methods with freshly prepared protoplasts. Briefly, 1 mL of the spore suspension (10^6^/mL) was inoculated in 50 mL of liquid M_3_S medium and incubated for 24 h at 28°C and 150 rpm. The fungal mycelium was filtered through a sterile funnel containing filter paper and washed 3 times with mycelial wash solution (MWS, 0.7 M KCl and 10 mM CaCl_2_). Approximately 100 mg of mycelial tissue was resuspended in a 50 ml Eppendorf tube containing 20 ml of enzyme solution that included 5 mg/ml of Lysing Enzymes (Sigma, L1412) and 2 mg/ml cellulose (Sigma, C8546) that were dissolved in MWS. After 2 h of incubation at 28°C with shaking at 70 rpm, the protoplasts were collected by filtration through three layers of sterile lens wiping papers. The concentration of the protoplasts was then adjusted to 10^7^–10^8^/mL of STC solution (1.2 M sorbitol, 10 mM pH 7.5 Tris–HCl, 10 mM CaCl_2_). One part SPTC (60% PEG4000 prepared in STC) was added to 3 parts protoplast solution, which was then aliquoted (100 μL each) into Eppendorf tubes. Five microliters heparin sodium (5 mg/mL) and 1 μg *Not* I digested plasmid was added to each tube, mixed thoroughly by tapping the bottoms of the tubes, and incubated on ice for 30 min. Another 1 mL SPTC was added and incubated for 20 min at room temperature. The protoplasts were mixed with 150 mL solid REG agar medium, incubated at 60°C, and quickly poured into ten Petri dishes. The plates were incubated at 28°C for 12–14 h, and then were covered with 10 mL 1% agar that contained 200 ng/ml hygromycin B and incubated for 3–5 days. The resistant colonies were regrown as single-spore colonies on M_3_S agar containing 150 mg/L hygromycin B and DNA was extracted for PCR confirmation. The primer pairs 5′F_end_Control with 925 and Hyg32 with 3′R_end_Control were used to verify correct integration within the genomes of the resistant transformants for the gene replacement stains. The complemented strains were screened using 150 ng/ml phleomycin (Sigma, P9564) and the expression level of GCS was confirmed using qRT-PCR for the mutant strains and complemented strains. Single conidia were isolated to purify the gene disruption mutants or complemented strains. Southern hybridization analysis was performed to confirm single-copy genomic integration. Genomic DNA obtained from the WT and three deletion mutants was digested with the appropriate restriction enzyme pairs. The 5′ flanking region was used as a probe for DNA gel blot analysis.

### Growth and Spores Germination Assay

For the radial growth assay, hyphal disks from the WT and deletion mutants with a diameter of approximately 1 mm were removed from the growing edges of cultures incubated on PDA or M_3_S. The disks were excised using a cut 200 μl pipette tip that had an inner diameter of approximately 1 mm. The cultures were grown at 27°C and radial growth measurements were taken at 24, 48, and 72 h after the transfer of the mycelia disk to a new M_3_S or PDA plate.

To measure germination for all strains that we examined, 50 μl of a conidial suspension (10^6^ conidia per ml) from each isolate was spread onto a M_3_S agar plate and maintained at 28°C. After 6 to 10 h of incubation, conidial germination was observed in three microscopic fields for each of five cells per slide. Conidial germination and appressorium development were monitored in three microscopic fields for each of five replicates using a 40× Olympus microscope CX41 (Olympus, Singapore). Conidia were considered to have germinated when the germination tube was at least three times longer than the conidia. The germination of the WT strain was set to 100% and was used for comparison to the mutant strains.

### Fruit Inoculation

Freshly harvested tomato fruit from a local market was used for pathogenicity assay. Conidia from the various *C. gloeosporioides* strains collected from a culture grown in mung bean broth (MBB) were inoculated onto peel-wounded tomato and mango fruit with a nail and placement of 8 μl of conidial suspension (10^6^/mL). Approximately 10 different fruit were used for each treatment. After inoculation, the fruit were incubated at 25°C and 95% relative humidity in covered plastic containers containing wet paper towels until symptoms were observed. The inoculation assay on avocado mesocarp was performed as previously described (Bi et al., 2017). The average decay diameter and the statistical analysis are given. The inoculation experiments were repeated three times and one representative experiment is presented.

### RNA Isolation and qRT-PCR Analysis

RNA was isolated from approximately 100 mg samples of frozen mycelia using the Eastep^TM^ Total RNA Extraction Kit (Promega, Beijing, China). The reverse transcription reaction was performed using 1 μg of total RNA using the PrimeScript^TM^ 1st Strand cDNA Synthesis Kit (TAKARA, Dalian, China), and the samples of cDNA were diluted 1:8 with ultrapure distilled water. Quantitative real-time PCR was performed using the StepOnePlus System (AB, Applied Biosystems) as previously described (Bi et al., 2017). PCR amplification was performed using 3.4 μl of diluted cDNA template in a 10 μl reaction mixture containing 5 μl of reagents from the SYBR-Green Amplification kit (TAKARA, Dalian, China) and 300 nM primers. PCR was carried out using the following cycling program: 30 s at 95°C, followed by 40 cycles of 95°C for 5 s and 60°C for 30 s. The samples were subjected to melting curve analysis, which observed efficiencies close to 100% for all primer pairs. The 18S gene of *C. gloeosporioides* was used as an internal control. The relative expression levels for each gene were analyzed using the 2^–ΔΔCt^ method (Livak and Schmittgen, 2001). The experiment was repeated at least three times with similar results. The result of one representative experiment are presented. The primer sequences were designed using Beacon Designer 7.0 software and are listed in Supplementary Table S1. The statistically significant differences within the results was performed with *t*-test.

### Transcriptome Analysis

Conidia (1 × 10^6^ spores/ml) from either the WT or a specific mutant were cultured in M_3_S liquid medium at 150 rpm for 72 h. The mycelia were collected and washed thoroughly with sterile distilled water, vacuum lyophilized, and vigorously ground into a fine powder using liquid nitrogen. The total RNA were isolated using the TRIzol Kit (Invitrogen) according to the user manual; the NanoDrop spectrophotometer and an Agilent 2100 Bioanalyzer were used to assess quality of the total RNA. The construction and sequencing of sequencing libraries were performed by Genebang (Chengdu, China) using the Illumina HiSeq × 10 platform.

### Extraction and Purification of GlcCer and Sphingolipid Analysis

Conidia (500 μl, 1 × 10^6^ spores/ml) from either the WT or a specific mutant were cultured for 3 days in M_3_S liquid medium at 150 rpm. The total sphingolipids were extracted from 1 g of mycelial powder via ultrasonic extraction in 10 mL of isopropanol:water:ethyl acetate (30:10:60, by vol) for 10 min in a water bath at 60°C; 100 μl of 1 μM C12 Glucosylceramide (d18:1/12:0), Sphingosine (d17:1) and C12 Ceramide (d18:1/12:0) (Avanti Polar Lipids, Alabama, United States) was added as an internal standard for idenfication of GlcCer, LCB, and ceramides, respectively. After centrifugation at 5000 rpm for 10 min, the upper phase containing GlcCer was dried using nitrogen and then dissolved in 500 μl methanol. The samples were injected into a Shimadzu UFLC-XR coupled with a hybrid quadrupole time-of- flight mass spectrometer (AB SCIEX Triple TOF 5600+)and gradient-eluted from a Phenomenex Luna C8 column (150 mm × 2.0 mm, 3 μm). The peaks corresponding to the target analytics and internal standards were collected and processed using the software MultiQuant (AB SCIEX). The components of the sphingolipids were determined as previously described (Markham and Jaworski, 2007).

## Results

### Expression Level of GCS During Infection and Identification of the GCS Gene in *C. gloeosporioides*

**FIGURE 1:**
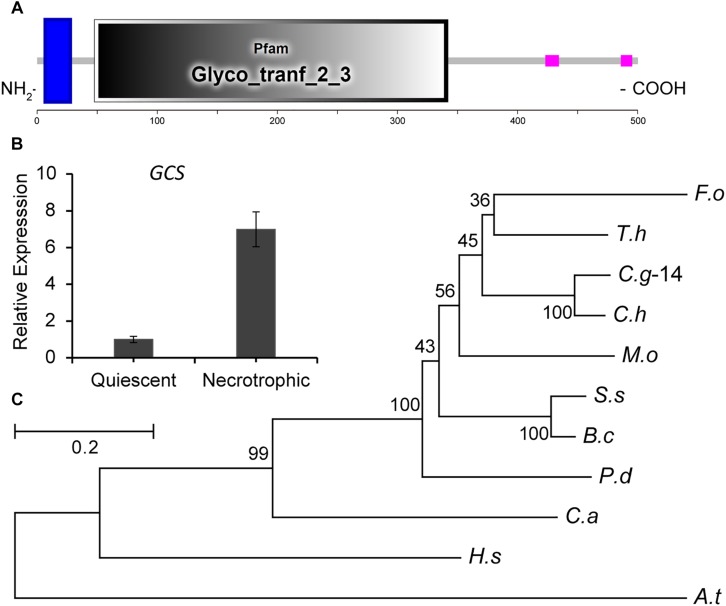
Functional domain identification, phylogenetic tree, and expression analysis of *CgGCS*. **(A)** Predicted domains of *C. gloeosporioides* GCS enzymes. SMART diagrams illustrate putative motifs and their locations in the amino acid sequences (represented by the numeric scales). Transmembrane and low complexity regions are indicated by blue and pink bars, respectively. Pfam domains and their predicted functions are represented by gray boxes. **(B)** Expression analysis of *CgGCS*. **(C)** Phylogenetic tree of putative CgGCS proteins identified in different organisms. *Fusarium oxysporum* f. sp. *lycopersici* (*F. o, EWZ93636.1*); *Arabidopsis thaliana* (*A. t, At2g19880*); *Colletotrichum gloeosporioides*-14 (C. g-14, EQB52767.1); *Magnaporthe oryzae* 70-15 (M. o, XP_003710106.1); *Homo sapiens* (H. s, BAA09451.1); *Candida albicans* SC5314 (C. a, XP_722809.1); *Penicillium digitatum* Pd1 (P. d, EKV12123.1); *Colletotrichum higginsianum* (C. h, CCF34817.1); *Trichoderma harzianum* (T. h, KKP00943.1); *Sclerotinia sclerotiorum* 1980 UF-70 (S. s, EDN96599.1); *Botrytis cinerea* BcDW1 (B. c, EMR84871.1).

During the infection of tomato fruit by *C. gloeosporioides*, the expression of *CgGCS* exhibits several-folds greater accumulation during the necrotrophic stage compared to the quiescent stage, which raised our interest in identifying its role in virulence (Figure 1B). Through a BLASTp search, one homolog (TCONS_00007676, contig01842, referred to here as *CgGCS*) was found to be homologous to the GCS genes in *P. digitatum* (EKV12123.1) and *Magnaporthe grisea* (XP_003710106.1), with 49% and 56% amino acid identity, respectively (Table 1). Sequencing analyses indicated that CgGCS contains 525 amino acids with an estimated molecular mass of 58.64 kDa and an isoelectric point (pI) of 8.25. The CgGCS protein contains an N-terminal transmembrane domain and a conserved Glyco_tranf_2_3 domain, and contains D1, D2, D3, and Q/RXXRW motifs that are essential for the enzymatic activity of mammalian GCS (Figure 1A and Supplementary Figure S1) (Leipelt et al., 2001). A phylogenetic tree of GCS homolog proteins that have been identified in various fungal genomes is shown in Figure 1C. The phylogenetic relationship of CgGCS to other GCS proteins revealed that GCS proteins in filamentous fungi are separate from those of *Candida albicans*, with *CgGCS* being most similar to that of other *Colletotrichum* species and most unlike those of the human and *Arabidopsis* (Figure 1C). The most distantly related sequences in fungi are still within 32% identity (Supplementary Table S1), which indicates that CgGCS proteins are well-conserved in fungi.

**TABLE 1:** Sequence identity of *CgGCS* with GCS in other organisms.

Identity	*H. sapiens* (BAA09451.1)	*A. thaliana* (At2g19880)	*C. albicans* (XP_722809.1)	*P. digitatum* (EKV12123.1)	*M. oryzae* (XP_003710106.1)	*C. higginsianum* (CCF34817.1)
*C. gloeosporioides* (EQB52767.1)	22%	5.33%	32%	49%	56%	79.6%

### Creation of GCS-Mutant Strains and Complemented Strains for *C. gloeosporioides*

**FIGURE 2:**
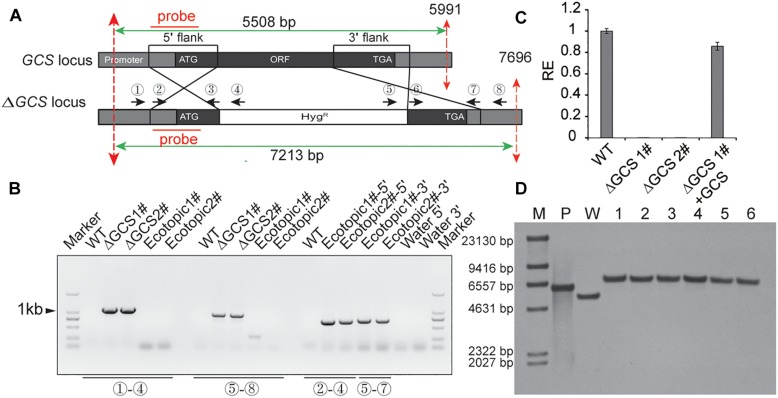
Generation of *CgGCS* mutant strains. **(A)** Replacement strategy used for deletion of *CgGCS*. The 5′ and 3′ flanking sequences were amplified using primer pairs 2 & 3 and 6 & 7, respectively. A double crossing-over homologous recombination event resulted in the replacement of the original *GCS* sequence with the *GCS* 5′ flank-Hyg-*GCS-*3′flank cassette. **(B)** PCR analysis of the wild-type (Musunuru et al., 2012) strain, ectopic colony (ectopic), and independent *GCS*-disruption colonies (Δ*GCS*). Primer 1 (Supplementary Table S1), which flanked a position upstream of the *GCS*: HYG region, and reverse primer 4 (Supplementary Table S1), which was located on the hygromycin cassette, were used to verify *GCS* gene replacement at the 5′ locus. Primer 5 (Supplementary Table S1), from the hygromycin cassette, and primer 8 (Supplementary Table S1), which flanked the *GCS*: HYG region, were used to verify *GCS* gene replacement at the 3′ locus. Primers 2 and 4 (Supplementary Table S1) and Primers 5 and 7 (Supplementary Table S1) were used as a positive control for the ectopic strains and to confirm random integration of the *GCS*:HYG cassette. **(C)** Expression of *GCS* in the WT strain compared to that of the ectopic-integration control, the *GCS* mutant strains and complemented strains, as measured by qRT-PCR. Cultures were grown in M_3_S-rich medium for 4 days prior to RNA extraction. The relative expression values obtained using qRT-PCR are normalized against that of 18 S rRNA. Expressed as the average ± SD of three replicates. **(D)** Southern Blot analysis. Genomic DNA from the WT, positive plasmid and ΔCgGCS strains was digested with *Bgl*II, whose restriction sites are shown by dotted red lines. 0.3 kb from each 5′ element region was used as the amplification probe, which resulted in an approximately 7.2 kb for all mutant strains but not for the wild type and confirmed gene disruption. M: marker; P: plasmid control; W: wild-type; 1–6: mutant strains.

To analyze the role of GCS in *C. gloeosporioides*, we created GCS deleted mutants using a gene replacement strategy (Figure 2). The GCS gene locus was inserted with a hygromycin-resistance cassette via homologous recombination (Figure 2A). The putative mutant clones were initially identified by PCR screening to verify the integration of the GCS-5′-Hyg-GCS-3′-cassette at the GCS locus (Figure 2B) using primer pair 1 and 4 and primer pair 5 and 8. As a result of correct integration, the PCR amplification produce a 941 bp amplicon and an 896 bp amplicon at the 5′ terminus and 3′ terminus, respectively, in *ΔCgGCS*1# and *ΔCgGCS*2#, but not in the WT and ectopic strains, suggesting the correct replacement of the GCS locus with hygromycin-resistance cassette. The amplification with primer pairs (2 and 4, 5 and 7) produce PCR products for the ectopic strains, and no PCR products was observed for the WT strain, indicating that random insertion of the hygromycin-resistance cassette occurred (Figure 2B). One round of single-spore purification was performed for excluding heterokaryons. Amplification of an internal fragment from GCS with qRT-PCR shown that no *GCS* transcript is observed, suggesting the complete deletion of GCS in the mutants (Figure 2C). To confirm if reintroduction of *CgGCS* was able to recover all observed mutant phenotypes, the plasmid pDEST phleo: GCS, which contained the native promoter, *CgGCS* genomic sequence and terminal sequence, was introduced into one of the deletion strains. The transformants were determined for phleomycin (120 μg/ml) resistance. QRT-PCR analysis confirmed the expression of GCS transcript in the complemented strains using (Figure 2C). In addition, six mutants were subjected to southern blot analysis using a probe specific to the 5′ flank of *CgGCS* to confirm that no ectopic integration was introduced (Figure 2A). As expected the positive plasmid and wild-type strain Cg-14 showed a 6.0-kb and 5.5-kb hybridizing band, respectively, and the six deletion strains had a 7.2-kb band (Figure 2D). These results shown that the six strains had a single copy insertion of CgGCS. Therefore, one representative deletion strain and one complemented strain were selected for further investigation.

### CgGCS Mutants Exhibited Reduced Radial Growth and Germination and Decreased Conidia Production

**FIGURE 3:**
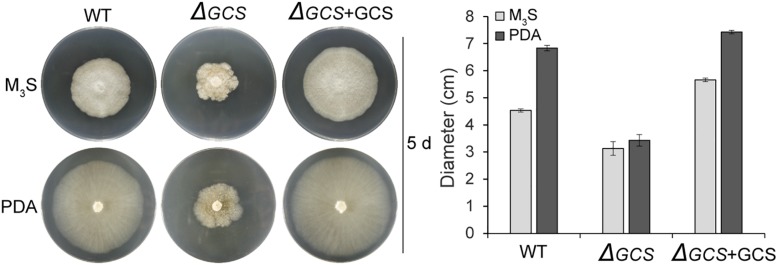
Radial growth assay on M_3_S-rich and PDA agar plates. Approximately 2 × 10^4^ conidia were spotted onto M_3_S or PDA agar plates. Plates were incubated at 28°C for 3 days. All experiments included two replicate plates and were performed at least three times with similar results. Bar = 10 mm.

**FIGURE 4:**
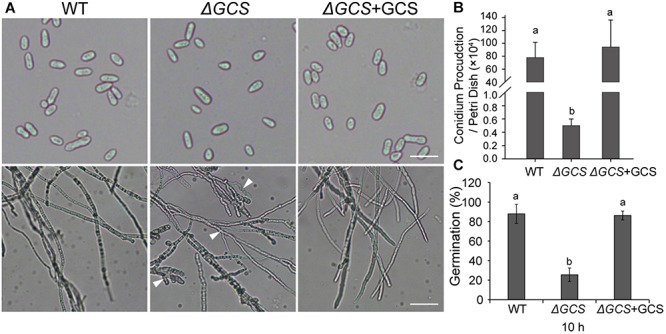
Phenotype analysis of wild type, mutant, and complemented strains. **(A)** Phenotype of spores and hyphae from each strain. **(B)** Conidia production in wild type, mutant, and complemented strains incubated for 7 days on M_3_S-rich agar plates. **(C)** Statistical analysis of the germination rate of each strain. The spores were spread on M_3_S plates and examined microscopically after 10 h. Arrows indicate wavy hyphae. Fisher’s Protected Least Significant Difference (PLSD) test was used to assess any statistically significant differences in the results.

We assessed the growth and colony morphology of the *ΔCgGCS* mutant when cultured in various media, including M_3_S and PDA media. Compared to the WT Cg-14 and complemented strains, the *ΔCgGCS* mutant grew much slower and exhibited denser vegetative hyphae (Figure 3). Examination under a microscope revealed that the mutant produced wavy hyphae with increased branching (Figure 4A). Conidiation assays showed that the *CgGCS* mutant was almost unable to produce spores on M_3_S agar plates or in M_3_S liquid medium (Figure 4B). This was also confirmed by using microscopic examination (data not shown). We attempted to use mung bean broth (MBB), which was used for spore production in *F. graminearum* (Hellin et al., 2018), as a second medium. In this liquid medium, the ΔCgGCS strain produced normal spores, and no morphological defects were observed in the conidia of the wild-type, mutant and complemented strains (Figure 4A). In addition, compared to the WT and complemented strains the rate of germination in mutants was greatly reduced after 10 h-inoculation, when they were cultured on M_3_S-rich agar plates in the dark (Figure 4C). These results indicate that *CgGCS* may be involved in regulating conidiation, spore germination and hyphal polar development in *C. gloeosporioides.*

### Disruption of CgGCS Affects Sphingolipid Profiles

**FIGURE 5:**
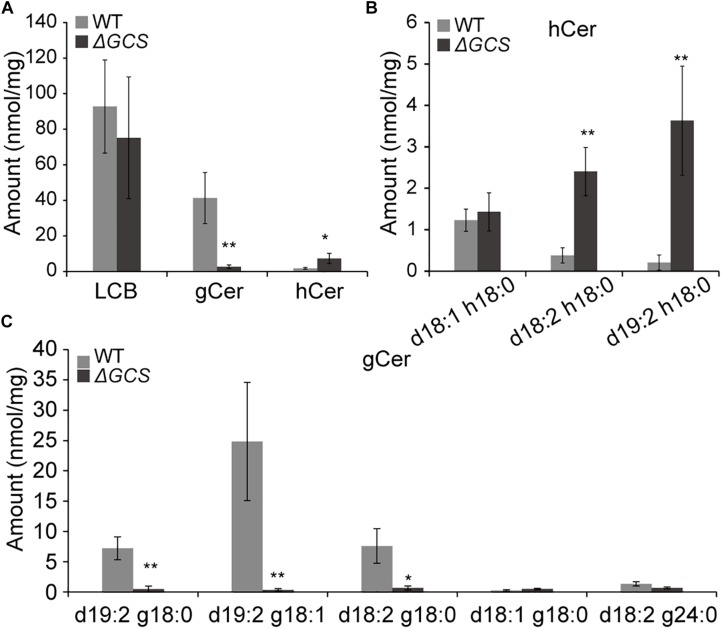
Sphingolipid content analysis for WT and mutant strains. Sphingolipids were extracted from mycelia grown on M_3_S medium for 3 days. The amounts of free long chain base (LCBs), main glucosylceramides and main hydroxyceramides in the wild-type and mutant strains were quantified after extraction, separation, and identification by HPLC coupled with electrospray ionization tandem mass spectrometry as described in the section “Materials and Methods.” The values indicate the absolute amounts of sphingolipids in the wild type and mutants. Significant differences between the mutants and the wild type at each time point were determined using Student’s *t*-test (*P < 0.05 and **P < 0.01). **(A)** Measurement of sphingolipids in mutant and wild type strains included total LCBs, glucosylceramides (gCer), and hydroxyceramides (hCer). **(B)** Measurement of main hydroxyceramide species in mutant and wild type strains. **(C)** Measurement of main glucosylceramide species in mutant and wild type strains.

To determine the sphingolipid profiles in the mutant strains, we compared the sphingolipid content of mycelia grown for 3 days in the WT and mutant strains. In total, we identified five glucosylceramides (GlcCer), five long chain bases (LCBs), and three different hydroxyceramides (hCer) in the hypha (Figures 5B,C). The five long chain bases were d18:0, d18:1, t18:0, t18:1, and d18:2. The three hydroxyceramides were d18:1 h18:0, d18:2 h18:0, and d19:2 h18:0 (Figure 5B), and the five glucosylceramides were d19:2 g18:0, d19:2 g18:1, d18:2 g18:0, d18:1 g18:0, and d18:2 g24:0 (Figure 5C and Supplementary Figure S2). Comparative analysis of the sphingolipid profiles disclosed that the total amount of hydroxyceramides was approximately 4-fold greater in knock-out strain than in the WT strain (Figure 5A), and the main hydroxyceramides that were increased in the mutant were d18:2 h18:0 and d19:2 h18:0 (Figure 5B). In the case of the glucosylceramides, their amounts were dramatically decreased in the mutant strain compared to those in the WT strain, and the main glucosylceramides that were decreased were d19:2 g18:0, d19:2 g18:1 and d18:2 g18:0 (Figure 5C). No significant changes were observed in the levels of LCBs (data not shown). In the mutant strain, the presence of increased levels of substrates and fewer products of glucosylceramide synthatase accumulation suggested that the CgGCS that we identified is a *bona fide* glucosylceramide synthase.

### CgGCS Mutants Show Impaired Virulence in Different Host Fruits

**FIGURE 6:**
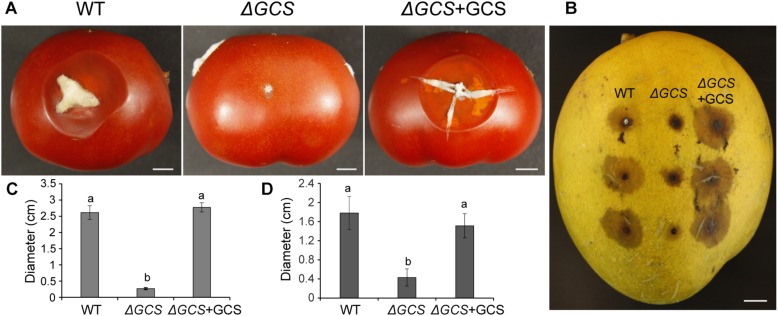
Pathogenicity assays of *C. gloeosporioides* strains on tomato **(A,C)** and mango **(B,D)** fruits. The fruits were inoculated with spores from wild-type, mutant and complemented strains and incubated for 5 days at 28°C. Bar = 1 cm. Letters indicate significantly different values using Fisher’s protected least significant difference, a *post hoc* multiple *t* test (*P* < 0.05).

To determine the contribution of CgGCS to *C. gloeosporioides* virulence, tomato and mango fruit were used as hosts for testing of the virulence of the *CgGCS* mutants. In tomato fruit, the mutant strain exhibited almost no pathogenicity 5 days after inoculation. In contrast, the WT and complemented strains resulted in the development of approximately 2.5 cm-diameter lesions at 5 days post inoculation (Figures 6A,C). In mango fruit, after 4 days inoculation the CgGCS mutants exhibit decreased virulence, small lesions were observed in the mutant-inoculated fruit, whereas in WT-inoculated fruit the lesion size was about 30 mm (Figures 6B,D). However, the complemented strain shown restoring virulence that be similar to that of the wild-type strain. The results indicated that deletion of *GCS* significantly impair the virulence of *C. gloeosporioides* in different fruit hosts.

### Transcriptome Analysis of WT and GCS Mutant Strains

**FIGURE 7:**
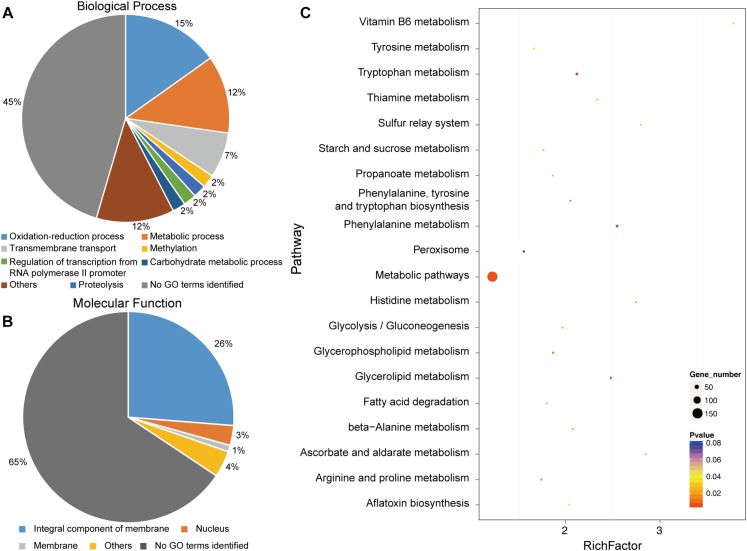
Functional categories and enriched pathways represented by the genes affected by CgGCS. The 581 differentially expressed genes were assigned to several categories of biological processes. **(A)** Molecular functional categories **(B)** according to the Gene Ontology Consortium database, and enrichment pathways **(C)**. KEGG analysis of the 20 most-enriched pathways. The coloring of the *q*-values indicates the significance of the enrichment factor. A circle indicates the target genes that are involved, and the size is proportional to the gene number. The Y-axis shows the name of the enrichment pathway. The X-axis represents the enrichment factor.

**FIGURE 8:**
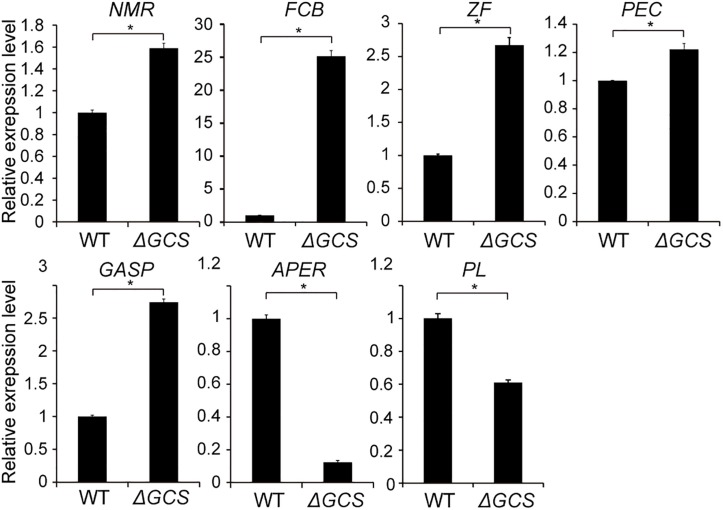
Expression level of seven selected genes in wild type and mutant strains. Gene expression values are relative to the average of the wild type (set as 1). The expression patterns of NmrA-like family protein (NMR, EQB59482.1); Amino acid permease (APER, EQB54120.1); Pectin lyase (PL, EQB57546); Glycolipid-anchored surface protein (GASP, EQB45424.1); Pectinesterase (PEC, EQB55391.1); C6 zinc finger protein (ZF, EQB55815.1); Fungal cellulose-binding domain-containing protein (FCB, EQB49523.1) were examined; 18S was used as an internal control. For each gene, the expression level in the WT was arbitrarily set to unity. The values are the average of two independent biological replicates. The error bars represent the standard deviation. The gene expression values are relative to the average for the wild type strains (set as 1). The experiments were repeated three times. *P < 0.05 according to Student’s *t*-test. Bars indicate standard deviation of means of three replications.

To identify putatively affected targets of the *GCS* mutation, the global gene expression patterns were compared between in the mutants and in the WT strain using transcriptome analysis. The results showed that 581 genes exhibited at least 2-fold differential expression between the WT and mutant strains (Supplementary File S1, Supporting Information). The 581 genes that were differentially expressed in the WT versus the Δ*GCS* strain were assigned to different biological and molecular functional categories (Figures 7A,B). Roughly half of the differentially expressed genes could not be matched to any functional category (Figure 7B). To validate the transcriptome data, reverse transcription-quantitative real time PCR (RT-qPCR) assay was performed to assure the differential expression of seven selected genes. The gene expression results of RT-qPCR show the same trends with that of transcriptome analysis, and the differential expression level exhibits some variation in the magnitude (Figure 8). Except no GO term identified genes encoding proteins potentially involved in oxidation and reduction processes were most abundantly represented among the differentially expressed genes (Figure 7A). Interruption of secondary metabolism in the mutant strain appeared to account in part for these differences. In addition, to further illuminate the biological interpretation of the differentially expressed genes we performed KEGG pathway analysis. The results indicated 367 genes is assigned to the top 20 enriched pathways (Supplementary File S2 and Figure 7C). According to the richness factor and gene number (151 genes; Supplementary File S2) “Metabolic pathways” was the most significantly enriched term, implying that mutation of GCS greatly impairs metabolism-related pathways in *C. gloeosporioides.*

## Discussion

Glucosylceramide is a sugar-coated sphingolipid. In this work, we presented a characterization of *CgGCS* in the postharvest pathogen *C. gloeosporioides*, with the goal of determining its importance for virulence. We also revealed the nature of glucosylceramide generated by GCS in *C. gloeosporioides*. Our results demonstrate that glucosylceramide is an important regulator of germination, development, sporulation and pathogenicity in *C. gloeosporioides.*

Previous reports indicate that mutation of GCS leads to decreased growth in the human pathogen *C. neoformans* (Rittershaus et al., 2006), and plant pathogens *P. digitatum* and *F. graminearum* (Ramamoorthy et al., 2007; Zhu et al., 2014). In our study, the growth rate of *CgGCS* deletion mutants was also significantly decreased compared to that of the wild type strain (Figure 3), which indicates the presence of conserved functions of GCS in different fungal pathogens. Furthermore, research of several fungal plant pathogens has shown that decreases of glucosylceramide in mutant strains greatly inhibits spore production and germination (Ramamoorthy et al., 2007; Zhu et al., 2014). In this research, the *CgGCS* deletion mutant exhibited almost no conidia production in normal growth conditions. Several studies have indicated that VeA/Ve1 or VelB is the key regulator of fungal conidiation (Merhej et al., 2012; Park and Yu, 2012). Our previous report indicated that a *Ve1* homolog may serve as an important regulator of conidiation in *C. gloeosporioides* (Bi et al., 2017). Unfortunately, the transcriptome data obtained from hyphae grown for 3 days in M_3_S medium showed that the expression level of the VeA/Ve1 (EQB45417.1) and VelB homologs (EQB56814.1) in *C. gloeosporioides* is comparable in the WT and mutant strains (Supplementary File S3). One possible explanation is that the transcriptome data does not represent materials obtained during the conidiation period of *C. gloeosporioides.* We believed that a deficiency in conidiation would contribute to impaired pathogenicity. Further researches are essential for complete disclose the function of GlcCer in conidiogenesis in *C. gloeosporioides*.

Glucosylceramide has been precisely characterized in many fungal species. In this study, in addition to GlcCer d19:2 g18:0 and d19:2 g18:1, which were identified by da Silva et al. (2004), another three GlcCer, d18:2 g18:0, d18:1 g18:0 and d18:2 g24:0, were also identified in *C. gloeosporioides*. The most abundant GlcCer, d19:2 g18:1, accounts for more than 50% percent of the total GlcCer in *C. gloeosporioides*, which is similar to the percentages that were determined in *A. nidulans* and *A. fumigatus* (Levery et al., 2002). The determination of the structure and species of GlcCer will aid in the development of specific antibodies that could be used to control specific fungal pathogens, since antibodies to GlcCer can effectively regulate fungal growth or differentiation (Rodrigues et al., 2000, 2007; Pinto et al., 2002).

Previous studies of plant defense responses found that fungal GlcCer also plays an important role in the pathogenicity of several plant fungal pathogens (Ternes et al., 2006; Zhu et al., 2014); in *F. graminearum*, the effect of FgGCS1 on pathogenicity was shown to be host-dependent (Ramamoorthy et al., 2007). In this study, we investigated pathogenicity in two different fruit hosts. Mutation of *CgGCS* greatly reduced virulence in both fruit hosts (Figure 6). We hypothesized that the reduced growth rate of the GlcCer-deficient strain also contributed to the pathogenesis of *C. gloeosporioides.* In addition, our transcriptome analysis indicated that “metabolic pathways” was the most significantly enriched term, which is consistent with the functioning of GlcCer as an important metabolite. Differential expression analysis indicated that a total of 13 proteolysis-related genes were differentially expressed; roughly two-thirds of these genes were upregulated in the WT compared with the Δ*GCS* strain (Supplementary File S1). Proteases secreted by fungal pathogens are reported to be associated with the degradation of host proteins resulting from plant-pathogen interactions (Jashni et al., 2015). For instance, the pectin lyase, which can catalyze the depolymerization of esterified pectin (Lara-Marquez et al., 2011), was upregulated in the WT compared with the Δ*GCS* strain (Figure 8). Among genes encoding proteins related to oxidation-reduction process, the mostly abundant part of differentially expressed genes, the expression of three putative cytochrome P450 genes was repressed in mutant (Supplementary File S1). Recent reports indicate that in *F. graminearum* mutation of cytochrome P450 greatly affect vegetative growth, particularly the deletion mutant exhibit ten-times less spore production compare to WT strain, and transcript levels of genes related to conidiation was greatly repressed in mutant (Shin et al., 2017), which may imply that the repression of cytochrome P450 in *C. gloeosporioides* also contribute to reduced growth and conidiation. In the genes that assigned to transmembrane transport a total of 20 major facilitator superfamily transporter (MFS transporter) genes were differentially expressed, and half of them were down-regulated in mutant, which may be involved in the phenotype impairment of mutant since MFS transporter paly important role on fungal growth, conidiation and full virulence in *Alternaria alternata* (Lin et al., 2018). In addition, an ammonium transporter (EQB50647) and two amino permease genes (EQB54120 and EQB43384) were also differentially expressed in WT vs. Δ*GCS*, which might affect secretion of small pH-affecting molecules because ammonia secretion related genes also modulate the pathogenicity in alkaline fungi *C. gloeosporioides* (Miyara et al., 2010). Indeed, for mutant strain the pH increase was greatly affected compare to WT strain when they was cultured in pH-induced medium (Supplementary Figure S3A).

Recently, several studies have revealed the important role of GlcCer/GCS in the regulation of alkali tolerance. A *C. neoformans* mutant lacking the glucosylceramide synthase (Gcs1) fails to replicate and grow at a neutral/alkaline pH and was found to lack virulence in a mouse model (Rittershaus et al., 2006). The growth of a *C. neoformans* strain was severely inhibited *in vitro* at a neutral/alkaline pH. For the plant pathogen *F. graminearum*, a Gcs1 mutant also exhibits severely reduced radial growth in a high pH solid medium (Ramamoorthy et al., 2007). Another study of *C. neoformans* showed that methylation of GlcCer is critical for its functioning during alkaline stress (Ternes et al., 2006). For *C. gloeosporioides*, previous studies have indicated that host-tissue alkalinization via ammonia accumulation is critical for colonization of *Colletotrichum* spp. (Miyara et al., 2010). In the present research, we compared the growth rates of the wild-type Cg-14 and Δ*CgGCS* strains in parallel by culturing them on M_3_S media with different pH values, which showed that they exhibited similar growth trends in M_3_S medium with different pH values (Supplementary Figure S3A). To further analyze the effect of *GCS* deletion on pH regulation in *C. gloeosporioides*, the fungus was grown in pH-induced medium (Miyara et al., 2010) at an initial pH 4. The results showed that an increase in the pH of the glutamate medium in the presence of mutant strains is apparently delayed compared to that of the WT strain (Supplementary Figure S3B), indicating that *CgGCS* affects the secretion of small pH-affecting molecules by *C. gloeosporioides*. In addition, we also examined the pathogenicity of WT and mutant strains on fruits with higher pH value. As expected the mutant strain still keep significantly reduced virulence compare to WT strain (Supplementary Figure S4), suggesting that reduced pathogenicity of GlcCer-deficient mutant don’t affect by host environmental pH. All of these results suggested that, in *C. gloeosporioides*, GlcCer does not contribute to environmental alkali tolerance but is involved in environmental pH regulation. This might contribute to virulence impairment on different fruit hosts. The underlying mechanism remains to be elucidated.

In mammals, because fungal GCS is structurally different from the mammalian homolog, GCS protein has been considered as a potential target for small molecule inhibitors that affect GlcCer synthesis (Del Poeta et al., 2014). Monoclonal antibodies (Rodrigues et al., 2000, 2007; Nimrichter et al., 2004) and plant or insect defensins (Thevissen et al., 2007, 2012; Tavares et al., 2008) targeted to fungal GlcCer were also used to control mammal fungal infections because of the vital role of GlcCer in the growth or virulence of fungi. In addition, previous reports have shown that spraying of long dsRNAs targeting the three fungal ergosterol biosynthesis genes can effectively control *F. graminearum* (Koch et al., 2016). The GCS gene is essential for the growth, sporulation and virulence of *C. gloeosporioides* and the sequence of the *CgGCS* gene shares low identity with that of the plant homolog; therefore, we attempted to spray a 360 nt-long dsRNA targeting the *GCS* gene in an attempt to control *C. gloeosporioides.* Tomato fruits were sprayed with GCS-dsRNA and then, 2 days later, were drop-inoculated directly into a sprayed area containing *C. gloeosporioides* spores. At three to five days post inoculation, the GCS-dsRNA-treated fruit exhibited lesions that were significantly smaller than those on control treatment fruits (Supplementary Figure S5), suggesting the potential application of spraying to control postharvest disease. From a more practical perspective, these results clearly indicate that we can study and develop new antifungal compounds with a broad spectrum of activity using GCS and GlcCer as excellent target candidates, because glucosylceramide synthase is highly conserved in many fungi but is different from the mammalian and plant homologs.

To summarize, GlcCer regulates growth, conidiation and pathogenicity by affecting the expression of a series of functional genes in *C. gloeosporioides*. These results will enhance our understanding of the mechanisms underlying the interaction between *C. gloeosporioides* and fruits and will facilitate the efficient control of anthracnose disease in fruits.

## Author Contributions

FB, GY, and CL designed the experiments. YH, BL, JY, and FB performed the experiments. QY, GD, OS, and TaD analyzed the experiment data. CH, ToD, and HG prepared the figures. FB wrote the draft of the manuscript. JY performed the sphingolipid analysis experiments for the fungi. All authors significantly contributed with reviewing and editing the manuscript.

## Conflict of Interest Statement

The authors declare that the research was conducted in the absence of any commercial or financial relationships that could be construed as a potential conflict of interest.

## Supplementary Material

FIGURE S1 Alignment analysis of the amino acid sequences of CgGCS and its homologs from other organisms using the *Clustal X* program.

FIGURE S2 The structure and fragmentations information of GlcCer in *C. gloeosporioides.*

FIGURE S3 The effect of CgGCS on the response to environmental pH **(A)** and alkalization **(B)** in *C. gloeosporioides.* **(A)** Growth assay of the wild type and mutant grown on agar plates with different initial pH values. M_3_S plates with different pH values were by adding either HCl or KOH. A 6 mm mycelia disk was placed in the center of the M_3_S plate and incubated at room temperature for 7 days. **(B)** Cultures were initially grown in M_3_S-rich medium and were then transferred to a second medium containing 1% glutamate (w/v) as a nitrogen source at pH 4, and the pH value was checked at different time points.

FIGURE S4 Decay development of WT and mutant strain on avocado fruit. Seven microliter spores (10^6^/ml) of WT and mutant strain were inoculated on 0.5-cm-diameter disks of sterile filter paper that placed on the peeled fruits before inoculation, and the fruits were incubated at 25°C under high humidity. Pictures were taken 4 days after inoculation. Bar = 1 cm. Significant differences between the mutants and the wild type were determined using Student’s *t*-test (**P* < 0.05), Bars indicate standard error.

FIGURE S5 *C. gloeosporioides* control on fruits inoculated with GCS-dsRNA. Tomato fruits were inoculated with GCS-dsRNA and TE (mock control). After 48 h, the fruits were drop-inoculated with 2 × 10^6^ conidia mL^–1^ of *C. gloeosporioides* onto the inoculated area and examined for decay lesions at 5 days post inoculation. Significant differences were determined using Student’s *t*-test (***P* < 0.01), Bars indicate standard deviation.

TABLE S1 Primers used in this study.

FILE S1 Detailed information of differential expressed genes.

FILE S2 The most enriched pathways that was identified for differential expressed genes.

FILE S3 Detailed information of gene expression between WT and mutant strain.
