# Supplementary figures and images for: CgGCS, Encoding a Glucosylceramide Synthase, Is Required for Growth, Conidiation and Pathogenicity in Colletotrichum gloeosporioides
Source: Front Microbiol. 2019 May 21;10:1016. doi: 10.3389/fmicb.2019.01016 (PMC6536669; doi:10.3389/fmicb.2019.01016)

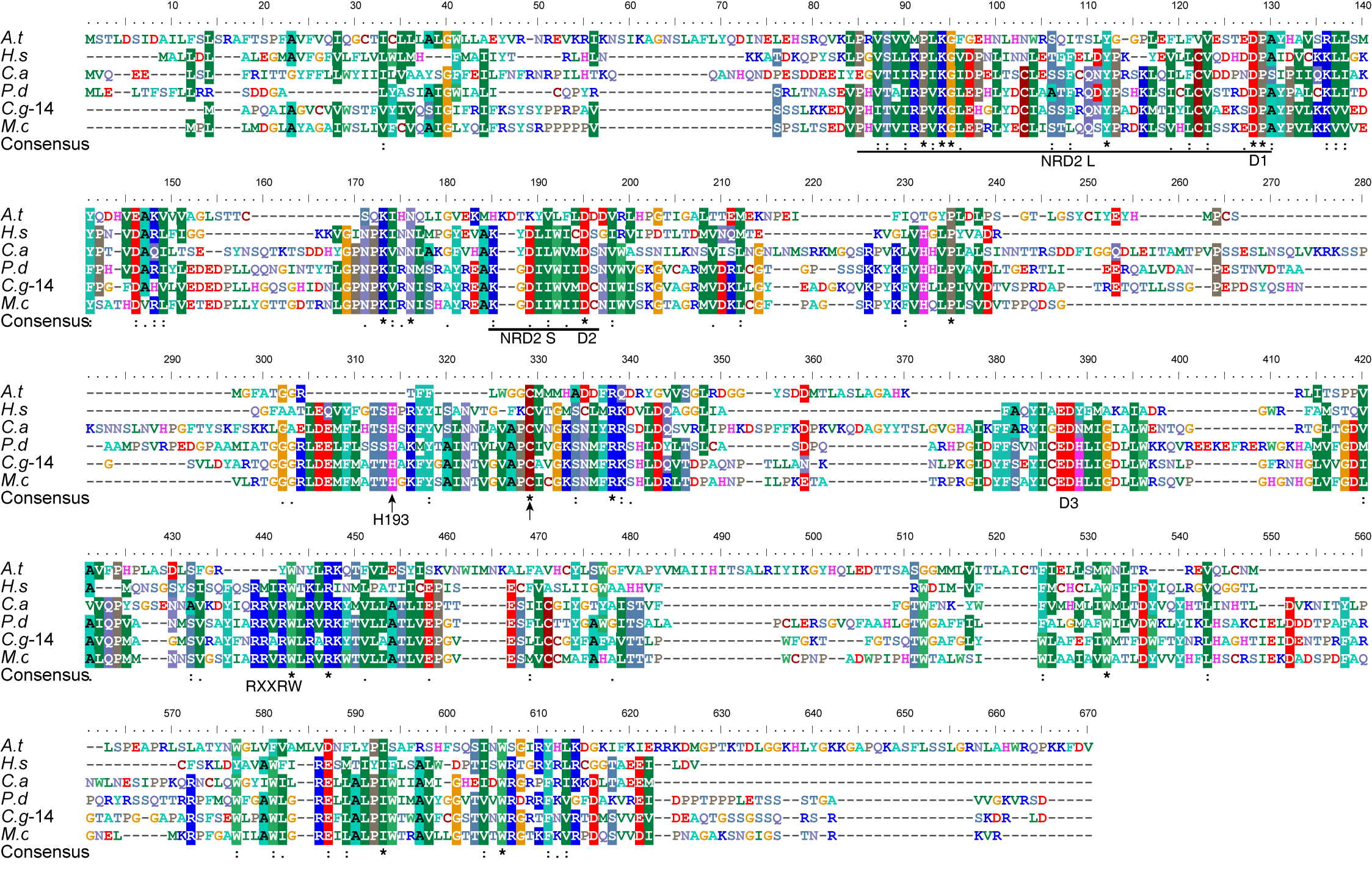

Supplement: FIGURE S1 — Alignment analysis of the amino acid sequences of CgGCS and its homologs from other organisms using the Clustal X program. [file Image_1.TIF]

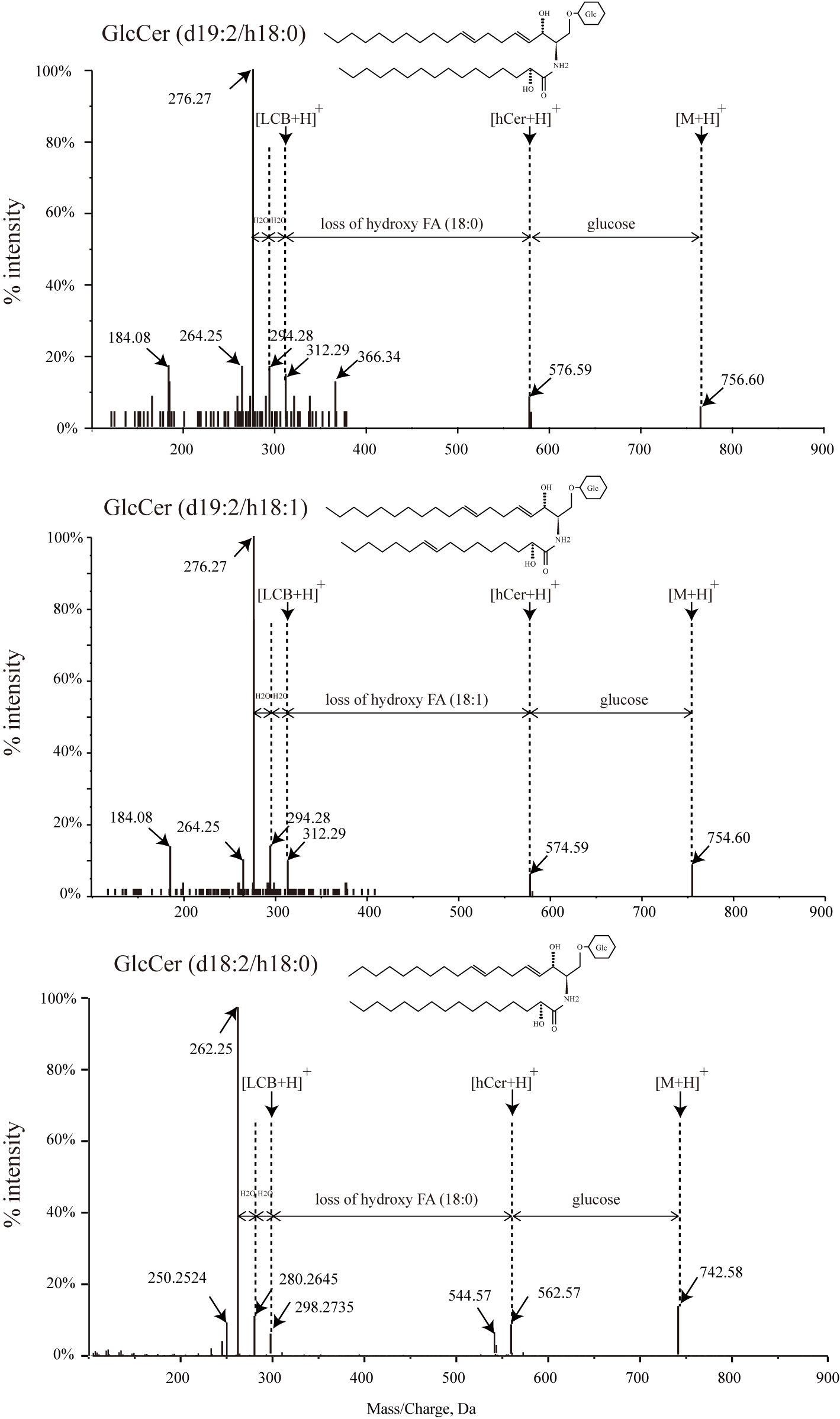

Supplement: FIGURE S2 — The structure and fragmentations information of GlcCer in C. gloeosporioides. [file Image_2.TIF]

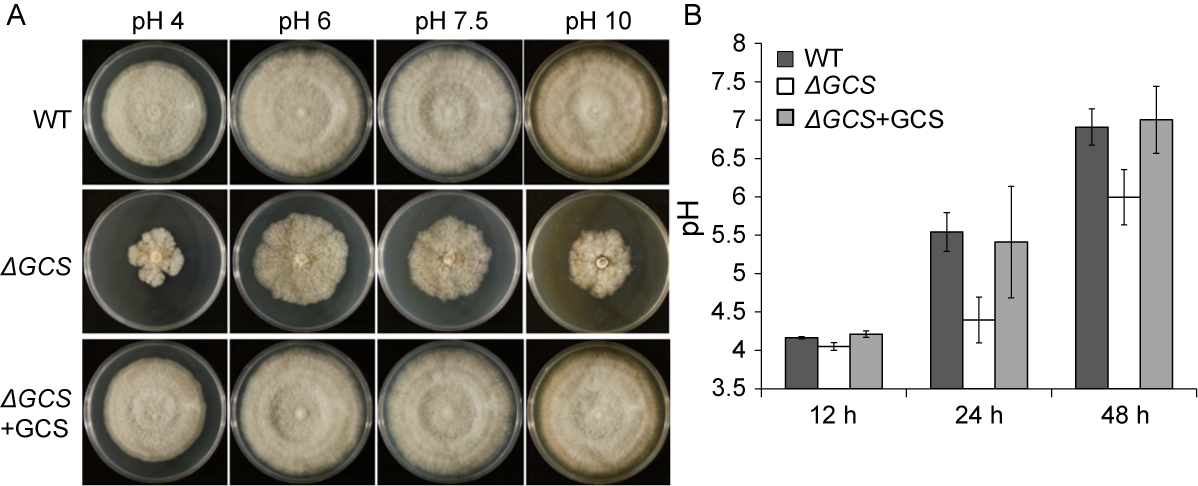

Supplement: FIGURE S3 — The effect of CgGCS on the response to environmental pH (A) and alkalization (B) in C. gloeosporioides. (A) Growth assay of the wild type and mutant grown on agar plates with different initial pH values. M3S plates with different pH values were by adding either HCl or KOH. A 6 mm mycelia disk was placed in the center of the M3S plate and incubated at room temperature for 7 days. (B) Cultures were initially grown in M3S-rich medium and were then transferred to a second medium containing 1% glutamate (w/v) as a nitrogen source at pH 4, and the pH value was checked at different time points. [file Image_3.TIF]

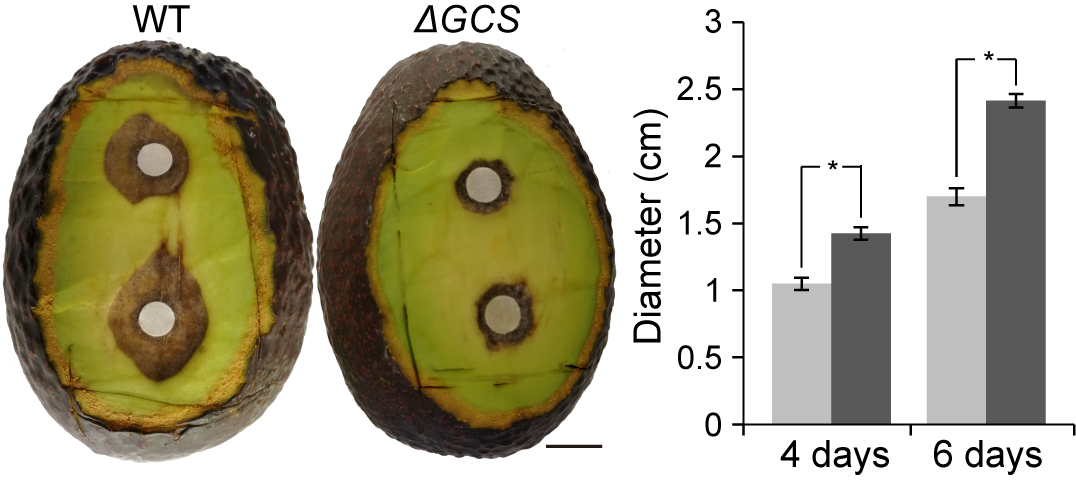

Supplement: FIGURE S4 — Decay development of WT and mutant strain on avocado fruit. Seven microliter spores (106/ml) of WT and mutant strain were inoculated on 0.5-cm-diameter disks of sterile filter paper that placed on the peeled fruits before inoculation, and the fruits were incubated at 25°C under high humidity. Pictures were taken 4 days after inoculation. Bar = 1 cm. Significant differences between the mutants and the wild type were determined using Student’s t-test (*P < 0.05), Bars indicate standard error. [file Image_4.TIF]

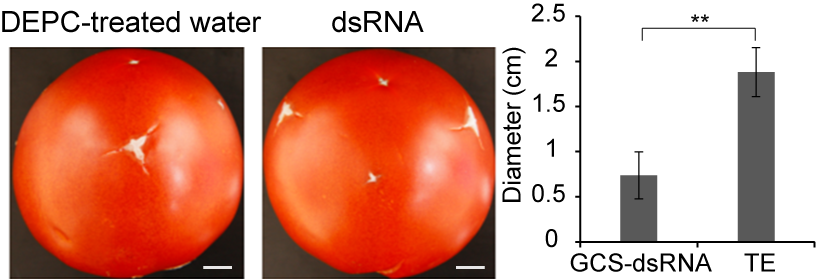

Supplement: FIGURE S5 — C. gloeosporioides control on fruits inoculated with GCS-dsRNA. Tomato fruits were inoculated with GCS-dsRNA and TE (mock control). After 48 h, the fruits were drop-inoculated with 2 × 10^6^ conidia mL–1 of C. gloeosporioides onto the inoculated area and examined for decay lesions at 5 days post inoculation. Significant differences were determined using Student’s t-test (**P < 0.01), Bars indicate standard deviation. [file Image_5.TIF]
