# Supplementary material for: CgGCS, Encoding a Glucosylceramide Synthase, Is Required for Growth, Conidiation and Pathogenicity in Colletotrichum gloeosporioides
Source: Front Microbiol. 2019 May 21;10:1016. doi: 10.3389/fmicb.2019.01016 (PMC6536669; doi:10.3389/fmicb.2019.01016)
Supplement: TABLE S1 — Primers used in this study. [file Table_1.docx]

Table S1. Primers used in this study

| Primer | Forward | Reverse |
| --- | --- | --- |
| Cg18 S | AGCGGAAAGCCTCGCAGT | TGTCGTTACCATCTCGACCCA |
| GCS | ATTGCTGGCGTATGCGTT | CTAATCTAAACGGTCTTTACTTCTT |
| GCScomp | GGGGACAAGTTTGTACAAAAAAGCAGGCTAGAGATTGGATTACTCGGATAGAAG | GGGGACCACTTTGTACAAGAAAGCTGGGTGCAAAGAAATCGGCGAAGAC |
| attB_GCS_5  attB_GCS_3 | GGGGACAACTTTGTATAGAAAAGTTGGCGGCCGCCTCCCGTCAACTTCAGACTCTT  GGGGACAGCTTTCTTGTACAAAGTGGCTGCTCTGCTGTGGTTATTTTG | GGGGACTGCTTTTTTGTACAAACTTGCTCTTCTCGGCTACACAGAGGT  GGGGACAACTTTGTATAATAAAGTTGGCGGCCGCTAATGGTTGAAAGGGAATGGAG |
| qGCS | GGAACATTAGTCGTGCCTACC | CGTCAGCCTCATAACCAAGAAG |
| GCS_5'C  GCS_3'C  925  Hyg32 | ATCCATTGTCGCTTGTTTCC  CTGAAGATCGTGTTCTGGTTTG  GCCTGGACGACTAAACCA  ACCTACTACTGGGCTGCT |  |
| NMR | GCTGGAGATGGTTCTTATGTTGT | TGTGGAGATGTTCCTCACTTGA |
| APER | CGTGGCTGGCTTGTTCTT | GATGCGGCGACTGTGATAC |
| PL | ATCGGTCGTCAGCATATTGTC | AGGTGTGGTGGATGTAGTTCT |
| GASP | GGTTACTCTGCTGCTGACATC | GCCTCCTTGAAGTTGGTGTT |
| PEC | CCTCTCAACGACCTCTTCCA | GCTCTTCTTGGCGGTGATG |
| ZF | TCATACGCACCTCGGAGAC | TCGGCAACCATCGCAATC |
| FCB | GGCACGGAGTTCTACTTCAAC | AGGACTTGGCGTTCTGGTA |
